# Mechanisms of *Moringa oleifera* Leaf Extract Influences Productive Performance, Immunity, Milk Composition, and Rumen Microbiota in Ruminants: A Review

**DOI:** 10.3390/vetsci13080821

**Published:** 2026-08-18

**Authors:** Mudathir Y. Abdulrahman, Nasir A. Ibrahim, Mohamed Osman Abdalrahem Essa, Saber Y. Adam, Raza Mohai Ud Din, Abdelkareem A. Ahmed, Rifat Ullah Jan, Hamdi Bendif, Nosiba S. Basher, Ahmed A. Saleh, Hosameldeen Mohamed Husien, Mengzhi Wang

**Affiliations:** 1 College of Animal Science and Technology, Yangzhou University, Yangzhou 225009, China; mudathir@duc.edu.sd (M.Y.A.); saber@duc.edu.sd (S.Y.A.); mh24136@stu.yzu.edu.cn (R.M.U.D.); dh23053@stu.yzu.edu.cn (R.U.J.); elemlak1339@yzu.edu.cn (A.A.S.); 2 Biomedical Research Institute, Darfur University College, Nyala P.O. Box 155, Sudan; aabdallah@buan.ac.bw; 3 Department of Biology, College of Science, Imam Mohammad Ibn Saud Islamic University (IMSIU), Riyadh 11623, Saudi Arabia; naabdalneim@imamu.edu.sa (N.A.I.); hlbendif@imam.edu.sa (H.B.); nsbasher@imamu.edu.sa (N.S.B.); 4 College of Veterinary Medicine, Yangzhou University, Yangzhou 225009, China; dh23054@stu.yzu.edu.cn; 5 College of Veterinary Medicine, Albutana University, Rufaa 22217, Sudan; 6 Animal and Fish Production Department, Faculty of Agriculture (Al-Shatby), Alexandria University, Alexandria 11865, Egypt; 7 State Key Laboratory of Sheep Genetic Improvement and Healthy Production, Xinjiang Academy of Agricultural Reclamation Sciences, Shihezi 832000, China

**Keywords:** immunomodulation, methanogens, propionate, rumen microbe, somatic cells

## Abstract

*Moringa oleifera* leaf extract (MOLE), including polysaccharides, polyphenols, amino acids and other extracts, are increasingly being used as feed additives in ruminant nutrition due to their high profiles of bioactive compounds. This comprehensive narrative review aims to review current research on their use in ruminant production regarding their productive performance, immune status, rumen microbiota and fermentation. Dietary supplementation with MOLE enhances growth rates and feed conversion efficiency, as well as immune status, milk yield and rumen microbiology, in ruminants by improving nutrient digestibility and metabolic efficiency. Additionally, MOLE exerts significant immunomodulatory effects, and studies indicate that immunoglobulins, antioxidants, cytokines, and enzymes reduce oxidative stress markers and incidence of subclinical diseases in dairy animals. Furthermore, supplementation with MOLE often increases milk fat and protein content while reducing somatic cell counts (SCCs) in milk. MOLE enhance the function of the rumen fermentation profile by increasing volatile fatty acid (VFA) production, especially propionate, and also regulating the stability of rumen pH. Additionally, it also selectively promotes microbial community diversity, enhances the population of cellulolytic bacteria and has a suppression function in protozoa and methanogens. Moreover, MOLE also contributes to improving fiber degradation and reducing the emissions of methane. In conclusion, MOLE is considered as a viable natural strategy to promote productivity, immune function, health, and environmental sustainability while decreasing methane production in ruminant production.

## Simple Summary

*Moringa oleifera* is a very fast growing tree with leaves rich in beneficial natural compounds. A simple extract from these leaves when added to the feed of cattle, goats and sheep provides several benefits. The animals gain weight and utilize feed better, and the dairy cows yield higher milk production with higher fat and protein levels, and fewer incidences of udder infection. Importantly, this natural supplement enhances the immune system of the animals so that they can remain healthier and with fewer medical treatments. The nutrient extract in the animals’ specially designed stomach (rumen) helps to restore a healthy balance of “good” microbes which ensure the breakdown of plant fiber, giving improved digestion and maintaining a balanced rumen environment. Concurrently, it lowers the amount of microbes producing methane—a powerful greenhouse gas that is linked to climate change. Overall, Moringa leaf extract is a promising and sustainable natural solution for modern agriculture, offering a potential approach for improving the growth, quality of milk, health of animals, and reducing harmful emissions from livestock.

## 1. Introduction

The rising cost of commercial feed concentrates underscores the significance of forage source and nutrient composition in dairy production [1]. Enhancing feed utilization efficiency and productive performance in ruminants, while simultaneously improving animal health and modulating the ruminal microbial ecosystem, remains a central challenge for researchers in ruminant nutrition and microbiology [2]. This sector also contends with broader constraints such as shrinking arable land, reduced pasture and crop areas, water scarcity, and climate change, which collectively limit feed availability [3].

In response, there is a growing global shift toward developing natural feed additives, including purified plant extracts (e.g., tannins and saponins), herbal blends, and essential oils. Among these, *Moringa oleifera* leaf extract (MOLE) offers distinct advantages. Biologically, MOLE contains a broad spectrum of bioactive compounds—polysaccharides, flavonoids, phenolic acids, and glucosinolates—that simultaneously enhance rumen fermentation, modulate immune responses, and reduce oxidative stress, a multi-target profile rarely achieved by single-compound additives [4].

Economically, *Moringa oleifera* (MO) trees grow rapidly on marginal lands with minimal water and fertilizer inputs, and leaves can be harvested locally in many tropical and subtropical regions, reducing dependence on costly imported feed additives and supporting low-input organic production systems [5]. Thus, MOLE represents not only a natural alternative to antibiotic growth promoters but also a cost-effective and sustainable option for both small-scale and large-scale ruminant producers [4]. The plant grows in many different soil types and usually reaches a height around 5–10 m, sometimes even up to 15 m, with a light feathery crown [6,7]. Furthermore, the plant is native to India and has a wide ranging list in Africa. This plant is extensively farmed in places like the Pacific Islands, the Caribbean, the Philippines, South Africa, Asia, Florida, and Latin America [8]. *Moringa oleifera* leaf meal (MOLM) is an environmentally friendly protein source for ruminants due to reducing bio methane and carbon dioxide production and characteristics of fermentation [5]. MO is very useful as feed supplement for animals since the leaves are highly nutritious [9]. Moreover, previous studies have shown that extracts of MO, *Jatropha curcas* (JC) and *Aloe vera* (AV) produced large reductions in in vitro methanogenic activity when these supplements were fed with a regular ruminant diet [10]. Although, dietary inclusion of MO and other plant extracts are promising strategies for mitigating the rumen methane production [11].

Furthermore, Moringa is rich in antioxidants and serves as valuable livestock feed, thanks to its high content of protein, vitamins, carotenoids, and polyphenols, along with minimal anti-nutritional factors [12,13]. Extracts from the Moringa plant could be highly effective natural dietary supplements for replacing antibiotic growth promoters in ruminant diets [14]. Several studies also indicate that supplementing diets with Moringa leaves or their extracts boosts dry matter intake, milk solids (both total and non-fat), milk fat content, daily milk yield, and energy-corrected milk yield in ruminants [15]. Due to its diverse bioactive compounds, Moringa holds promise as a potent source for new pharmaceutical agents. These could enhance livestock productivity, safety, and product quality while mitigating the risk of antibiotic resistance development [16]. Several studies have described the use of MOL as animal feed (ruminants) resulting in optimizations of protein synthesis by the rumen microbes. Moreover, several studies have been conducted which have given information on the effects of MOL on performance and quality of milk in goats and cows [17]. Overall, supplementation of diets of Nubian goats with MO extract significantly improved the milk yield by about 6% and energy corrected milk yield by 12%. Moreover, MOLE decreased both individual and total saturated fatty acids in milk by approximately 4.6–5.6%, while raising individual and total unsaturated fatty acids by roughly 11.5–13.9% [18]. However, the blood concentration of glutamic and pyruvic transaminase was increased significantly and urea-N and cholesterol concentrations were decreased significantly in goats fed MLM diets. Furthermore, milk yield and energy corrected milk were increased significantly in goats fed MOLM and the greatest increase was found in the group fed the M15 diet [19]. Adding MOLM to the diet markedly influenced rumen fermentation parameters: it reduced ruminal enzymes, ammonia-N levels, total protozoa counts, and the acetate: propionate ratio, but elevated acetic acid, propionic acid, butyric acid, and overall total volatile fatty acid concentrations [20]. Plants possessing bioactive products like essential oils, saponins and condensed tannins with antimicrobial properties may be used in ruminant production in order to reduce CH_4_ emissions and enhance fermentation efficiency [21]. Therefore, the aim of this review was to synthesize the available literature regarding the nutritional, immunological, and rumen-modulating impacts of MOLE in ruminant production systems. We hypothesize that MOLE, when strategically supplemented, acts as a multi-functional feed additive capable of concurrently increasing milk production, enhancing immune status, mitigating methane emissions, and improving overall growth performance in ruminants.

## 2. Nutritional Composition and Bioactive Components of MOL

MO has been recognized as containing a great number of bioactive and nutritional compounds [22]. Previous studies have reported that Moringa is abundant in all essential macro- and micronutrients, as well as a wide range of bioactive compounds, including terpenoids, polyphenols, flavonoids, glucosinolates, alkaloids, glycosides, and carotenoMids [23] (Table 1). Additionally, MOL contains a very high amount of protein compared to other leaves as it is consumed as food. MO also contains essential amino acids along with a high quantity of pro vitamin A [24]. MOL represents an outstanding nutritional source, providing high levels of protein, provitamin A (β-carotene), vitamins B and C, along with substantial amounts of essential minerals—particularly iron—and a well-balanced profile of essential amino acids, including methionine, cysteine, tryptophan, and lysine [25]. Moringa seeds contain a high amount of unsaturated fatty acids (82%) that is mainly composed of oleic acid [26]. This tree has recently gained application in both animal and human nutrition due to its exceptional nutrient density, ranking among the most nutrient-rich plants known to date. It provides substantial levels of digestible protein (20–35% of dry matter, DM), containing all essential amino acids, along with crude fiber (7–35% DM), crude fat (7–20% DM), minerals (8–11% DM), and vitamins [27]. As a result, MOLE is recognized as a bio-stimulant, rich in key nutrients such as minerals, fiber, proteins, sugars, free amino acids, vitamins, phytohormones—including zeatin (a cytokinin derivative), auxins, and gibberellins, as well as abundant antioxidants [28]. Furthermore, MOL is a notable source of alkaloids, carotenoids, saponins, isothiocyanates, phenolic acids, glucosinolates, and flavonoids. These compounds are present in higher concentrations compared to commonly consumed fruits, vegetables, and other food plants. This rich bioactive profile is sufficient to support immune function and combat reactive oxygen species (ROS) [29].

Phytochemicals are biochemical metabolites which are found in plants in nature and have nutritional value in human life. These metabolites include alkaloids, flavonoids, steroids, glycosides, gums, phenol, tannings, terpenes and terpenoids, which have been used as chemical substances for the development and manufacture of synthetic drugs [30,31]. Furthermore, several scientific articles have been published narrating the antioxidant properties of Moringa, which can be translated to its use as an anti-aging herb [32,33]. The most important bioactive compounds found in moringa seeds have demonstrated anti-microbial, antigenotoxic, anti-inflammatory, and anti-tumor promoting properties [34]. However, all leaf extracts exhibited significantly stronger antidiabetic and antimicrobial activity compared to the roots [35]. Additionally, the antibacterial activity of MO oil extract demonstrated effectiveness against all tested bacterial strains at varying concentrations, with the exception of *E. coli* and pneumonia-causing bacteria, which exhibited resistance to the seed oil extract at a concentration of 125 µL/mL [36].

**Table 1:** The different bioactive components that can be extracted from different part of MO.

No	Bioactive Components	Species	Part of Plant	References
1	Polysaccharide	MO	Leaves	[24]
2	Vitamin A, E	MO	Fresh leaves	[22]
3	Terpenoids, polyphenols, flavonoids, glucosinolates, alkaloids, glycosides and carotenoids	MO	All part of plant	[23]
4	Phenolic acids and flavonoids	MO	Leaves	[24,37]
5	Unsaturated fatty acids	MO	Seeds	[38]
6	Proteins and functional peptides	MO	Stem	[39]
7	Crude fat, fatty acids and minerals	MO	Root, seed, stem	[40]
8	Polyunsaturated fatty acids such as omega-3 and omega-6	MO	Leaves	[24]
9	Moringyne, alpha-phellandrene,	MO	Seeds	[41]
10	4-(alpha-L-rhamanosyloxy) benzyl isothiocyanates	MO	Seeds	[42]
11	Oligosaccharides and oxalate	MO	Leaves	[43]
12	Beta carotenes	MO	Flowers	[44]
13	Flavonoids, saponins	MO	Seeds	[45]

## 3. Impact of MOLE on Milk Production and Their Components

**Figure 1:**
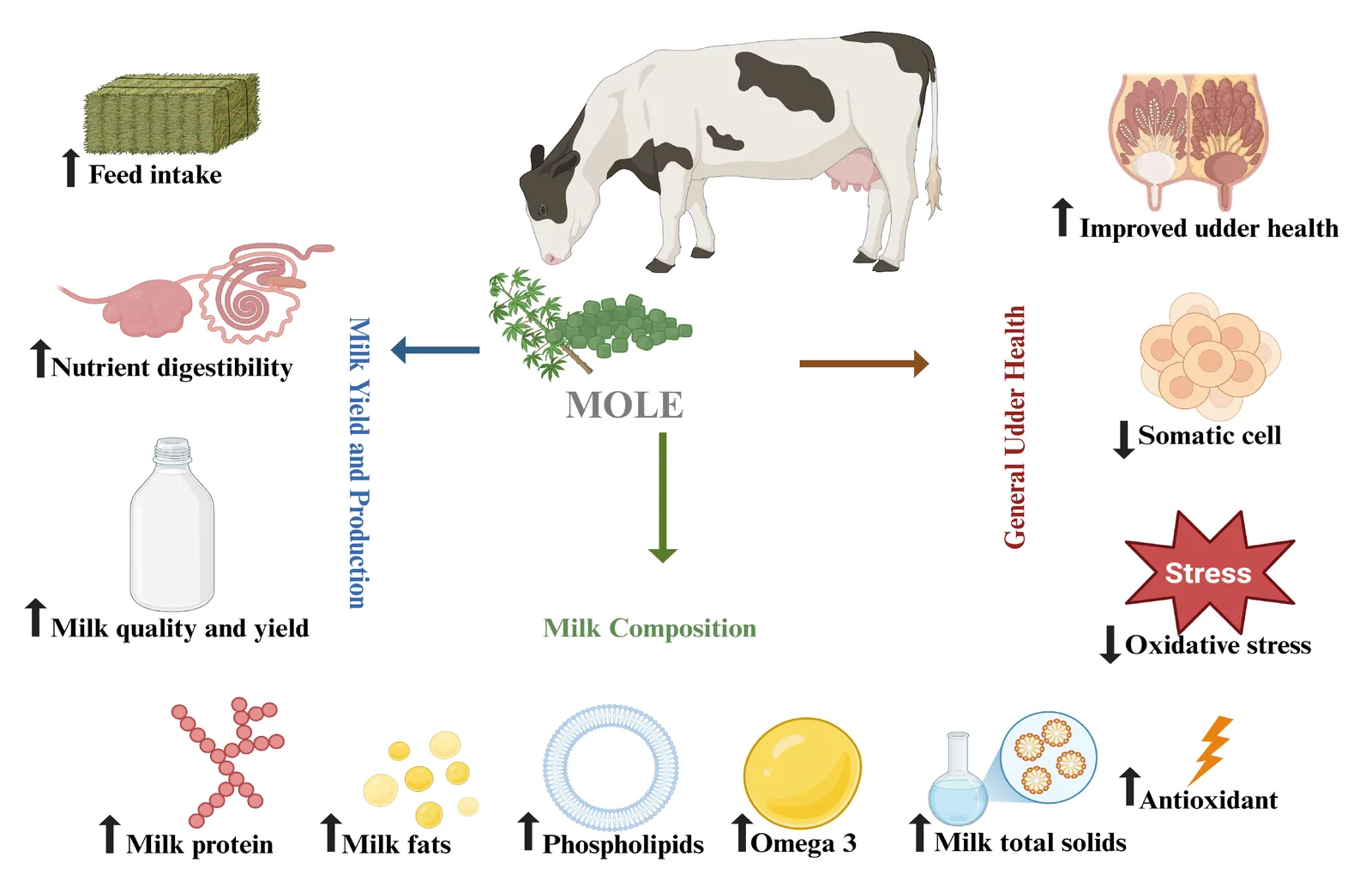
Schematic overview of the beneficial effects of dietary MOLE supplementation on feed utilization, milk production, milk composition, udder health, and oxidative stress mitigation in lactating dairy cows. The central illustration depicts cow consumption of MO (green leaves and powder). Supplementation enhances feed intake and nutrient digestibility (rumen/intestine icons), leading to improved milk yield and overall production. It supports general udder health (mammary gland depiction) and reduces SCC (somatic cell icon), indicative of lower subclinical mastitis risk and inflammation. Omega3: trimethylglycine (betaine), a methyl donor that alleviates oxidative stress and supports immune function. Arrows in the figure indicate the direction of change: upward arrows represent an increase in the corresponding parameter, while downward arrows represent a decrease.

MOL, seeds, and bark are readily accepted by cattle as part of their daily ration, with multiple studies reporting that supplementation with MOLE enhances milk yield and improves milk composition [3] (Figure 1). The magnitude of these effects varies by species, dose, and product form, as summarized in Table 2.

In goats, MOLE supplementation increased daily milk yield and energy-corrected milk, while decreasing saturated fatty acids and increasing unsaturated fatty acids [18]. Lactating goats fed MOLM at 15% of the diet showed increased milk yield and energy-corrected milk along with reduced blood urea-N and cholesterol [19]. Goats fed Moringa foliage also exhibited sustainable increases in milk yield and phospholipids, with elevated omega-3 fatty acids and a reduced n-6/n-3 ratio [46]. Furthermore, research has indicated minimal differences in feed intake and milk production across varying supplementation levels of MO; however, inclusion at 6% in the diet notably increased milk fat content [1].

In dairy cows, supplementing MOLM to transition dairy cows improved blood biochemistry profile, antioxidant status, and udder health. Milk composition and antioxidant capacity were enhanced, with colostrum showing higher fat, lactose, protein, and total solids. Colostrum IgG increased compared to the control group, and milk somatic cell count (SCC) was reduced [47,48]. Another report showed that supplementation of hydroalcoholic MOLE at doses of 20, 40 or 60 mL/ewe/d in lactating ewes did not have negative effects on milk yield and milk composition [15]. In contrast, one study found that in mid- and late lactation cows fed with MOLP, SCC, milk production, and plasma components concentrations were not affected [49].

In buffaloes, lactating buffaloes fed increasing levels of MOLP showed significant increases in total milk solids, total protein, and milk fat, whereas non-fat solids and lactose content decreased compared to the control group [50].

In ruminants overall, MO supplementation increased milk production in two out of three reviewed studies, showed variable effects on growth performance, and had no significant impact on iron status [51]. Additional investigations have demonstrated significant improvements in milk yield, energy-corrected milk, total solids, fat content, and energy concentration in animals receiving MO-supplemented diets [52]. One study found that MO foliage at 10% of total diet enhanced feed intake, milk production and its quality, nutrient metabolic profile, and immunity functions in Vrindavani cows [53]. Moreover, supplementation with hydroalcoholic extracts of MOL at doses of 20, 40 or 60 mL/ewe/d in lactating ewes had no negative effects on milk composition or milk production [15].

The improvements in milk yield and composition following MOLE supplementation arise from at least three interconnected mechanisms. First, MOLE may enhance rumen fermentation, increasing the production of VFA, particularly propionate and butyrate. Propionate serves as the primary gluconeogenic precursor in lactating ruminants, supporting glucose synthesis for lactose production, which in turn drives milk volume. Butyrate directly stimulates mammary epithelial cell proliferation and differentiation [20,54]. Second, the antioxidant compounds in MOLE (flavonoids, phenolic acids) reduce systemic oxidative stress, as evidenced by decreased malondialdehyde (MDA) and increased total antioxidant capacity (T-AOC) and catalase (CAT). Lower oxidative stress preserves the integrity of mammary alveolar epithelium and supports the synthesis of milk components, including fat and protein [55,56]. Third, the observed reduction in milk SCC (by up to 37% in transition cows [48]) indicates improved udder health, likely due to the direct antimicrobial activity of MOLE against mastitis pathogens and the immunomodulatory reduction in excessive inflammation [47,48]. The increase in unsaturated fatty acids and omega-3 content, alongside the decrease in saturated fatty acids, suggests that MOLE alters ruminal biohydrogenation pathways, possibly by inhibiting specific bacterial populations responsible for saturated fatty acid synthesis while promoting those that produce unsaturated isomers [18,46].

**Table 2:** Quantitative effects of MOLE/MOLM supplementation on milk production and composition in ruminants.

Species	Supplement Form	Dose/Level	Duration	Outcome Metric	% Change vs. Control	Citation
Nubian goats	MO extract	Not specified	Trial period	Daily milk yield	+6%	[18]
Nubian goats	MO extract	Not specified	Trial period	Energy-corrected milk	+12%	[18]
Nubian goats	MO extract	Not specified	Trial period	Saturated fatty acids (total)	−4.6 to −5.6%	[18]
Nubian goats	MO extract	Not specified	Trial period	Unsaturated fatty acids (total)	+11.5 to +13.9%	[18]
Lactating goats	MOLM	15% of diet	Trial period	Milk yield	Increased (significant)	[19]
Lactating goats	MOLM	15% of diet	Trial period	Energy-corrected milk	Increased (significant)	[19]
Transition dairy cows	MOLM	16.66 g/100 kg BW	Pre- + post-partum	Colostrum IgG	+42%	[47]
Transition dairy cows	MOLM	16.66 g/100 kg BW	Pre- + post-partum	Milk SCC	−37%	[48]
Lactating buffaloes	MOLP	Increasing levels	Trial period	Total milk solids	+8 to +12%	[50]
Lactating buffaloes	MOLP	Increasing levels	Trial period	Total protein	+5 to +7%	[50]
Lactating buffaloes	MOLP	Increasing levels	Trial period	Milk fat	+6 to +9%	[50]

## 4. Mechanism of Action of *Moringa oliefera* Leaf Extract (MOLE)

*Moringa oleifera* is a widely distributed tree with different species and common names across various countries (Table 3). Recent research has observed a notable increase in studies examining extracts from MOL prepared with aqueous, hydroalcoholic, or alcoholic solvents. These extracts demonstrate a broad spectrum of pharmacological activities. These include antioxidant, tissue-protective (hepatoprotective, nephroprotective, cardioprotective, testicular-protective, and pulmonoprotective effects), analgesic, antiulcer, antihypertensive, radioprotective, and immunomodulatory properties [57].

Important bioactive compounds underlying its therapeutic potential consist of various polysaccharides, along with vanillin, quercetin, gallic acid, chlorogenic acid, and ferulic acid [58] (Table 4). Furthermore, previous studies indicate that MOLE downregulates mRNA expression of leptin and resistin while upregulating adiponectin gene expression in obese rats compared to untreated controls [59]. The extract also demonstrates potent anti-inflammatory activity against both localized and systemic inflammation in an inverse dose-dependent manner. This effect is linked to decreased levels of nitric oxide and prostaglandin E2, mediated through the modulation of inducible nitric oxide synthase expression [60,61].

MOLE dose-dependently reduces the levels of CYP450 isoenzymes while simultaneously upregulating the Nrf-2 antioxidant system. This leads to increased levels of hepatic antioxidant enzymes [62]. Mechanistically, the activity of MOML against Dalton’s lymphoma (DL) cells is primarily mediated by inactivation of the MEK/ERK signaling pathway. Additionally, MOML-mediated suppression of DL tumor growth in vivo was linked to the induction of apoptosis and restoration of hematological parameters in DL-bearing mice [63].

The bioactive constituents of the plant exert anti-inflammatory effects via multiple mechanisms. These include: (a) inhibition of pro-inflammatory enzymes, such as suppression of cyclooxygenase (COX) and lipoxygenase (LOX) activities by flavonoids like quercetin and kaempferol; (b) modulation of cytokine production, wherein isothiocyanates regulate inflammation-associated signaling cascades (e.g., NF-κB pathway), thereby reducing the release of pro-inflammatory cytokines such as TNF-α and IL-1β; and (c) antioxidant activity [37].

The immunomodulatory effects of phytocompounds from traditional plants primarily involve macrophage activation, stimulation of phagocytosis and peritoneal macrophages, lymphoid cell stimulation, and the modulation of specific and non-specific cellular immune systems via multiple signaling pathways [64]. In wound healing, topical application of MOLE on excision wounds significantly accelerated healing. It promoted the expression of TGF, vascular endothelial growth factor (VEGF), and Type 1 collagen, while suppressing inflammatory markers such as IL-1β and TNF-α [65]. Collectively, these findings suggest that phytocomponents in various parts of the plant regulate diverse biological functions by modulating multiple molecular targets [66].

**Table 3:** Common names of MO and their distribution in different countries.

No	Scientific Name	Country	Common Name	References
1	*Moringa oleifera*	Worldwide	Drumstick Tree	[67]
2	*M. stenopetala*	Botswana, Kenya and Ethiopia	Cabbage tree	[68]
3	*M. peregrina*	Middle East and Red Sea region (e.g., Egypt, Saudi Arabia, Sudan)	Ben tree; wispy, Yasar tree; Wild drumstick tree	[69]
4	*Moringa oleifera leaf*	Pakistan, India, and Africa	Benzolive tree, Kelor tree, Mlonge tree, Marango tree, Saijihan tree, Sajna tree, and Mulangaytree	[70]
5	*M. rivae*	East Africa (Kenya, Ethiopia)	Swanjehro	[69]
6	*Moringa oleifera lam (Moringa pterygosperma G*	India, Bangladesh, and Afghanistan	Horseradish tree	[37]
7	*Moringa ovalifolia*	Pakistan	Sohanjan	[71]
8	*Moringa oleifera*	India and Africa	Miracle Tree or Tree of life	[24]
9	*Moringa oleifera*	Asia and Africa	Tree-foliage	[27]
10	*Moringa oleifera*	Northern Nigeria	Zogale	[72]

**Table 4:** Mechanism of action of different bioactive components extracted from MO.

Bioactive Components	Function	Mechanism of Action	References
Flavonoids and polyphenols	Anti-inflammatory	Inhibition of pro-inflammatory enzymes	[37]
Aqueous extract of leaves	Anti-hyperglycemic	Decreased blood glucose levels	[57]
Acetone extract of MOL	Antibacterial	Inhibit the growth	[73]
Ethanol extract of MOL (MOLE)	Regulated the Nrf-2 antioxidant system	Decreased the level of CYP 450 isoenzymes	[62]
Ethanol extract of MOL (MOLE)	Hypoglycemic effect	Improve glucose consumption	[74]
Ethanol extract of MOL (MOLE)	CNS depressant and anticonvulsant activities	Release γ-amino butyric acid (GABA)	[75]
Methanolic leaf extract (MOL)	Reducing glycemia	Increased insulin secretion and sensitivity	[76]
Ethanol extract of MOL (MOLE) (astragalin and isoquercetin)	Anticancer of colon cells	Down regulation of ERK1/2 phosphorylation	[77]
Ethanol extract of MOL (MOLE)	Improve the testicular structure	Germinal hyperactivity of cells	[78]
Ethanol extract of MOL (MOLE)	Therapeutic effectiveness against nephrotoxicity	Enhancement of the endogenous antioxidant system and a modulatory effect on specific inflammatory cytokines	[79]
Methanol leaf extract	Antibacterial	Growth inhibition	[80]
Aqueous extract of MOLE	Anti-allergic (anti-histamine)	Suppression of mast cell activation	[81]
Methanolic, hydro alcoholic and hydro alcoholic maltodextrin extracts	Bacteriostatic and bactericidal effects at concentrations of 0.5, 0.5 and 0.1 mg/mL	Alter membrane permeability	[82]

### Safety Considerations and Practical Inclusion Rates

Across the studies reviewed, MOLE and MOLM were supplemented at varying inclusion levels without any reported adverse effects on animal health, feed intake, or production performance. In lactating ewes, MOLE at doses up to 60 mL/head/day showed no negative impact on milk yield or composition [15]. In dairy cows, MOLM fed at 16.66 g/100 kg body weight during the transition period improved colostrum quality and reduced SCC without toxicity [47,48]. In goats, MOLM inclusion at up to 15–20% of the diet was well tolerated and associated with improved performance [19]. Regarding anti-nutritional factors, MOL contains only minimal levels of tannins, saponins, and phytates compared to other tropical forages, and at the concentrations present, these compounds contribute to its beneficial antioxidant and antimicrobial activities rather than causing harm [13]. Nevertheless, no dedicated dose–response or chronic toxicity trials have been conducted in ruminants to establish formal maximum safe inclusion rates. Future research should prioritize such studies to define upper dose limits and ensure long-term safety across different production stages and species.

## 5. Impact of MOLE on Immune Status

Moringa is rich in nutrients and bioactive compounds, making it a promising supplement for livestock feed. Its leaves, seeds, and bark are readily consumed by animals such as cows, sheep, goats, pigs, chickens, and rabbits [83]. Moreover, MOLE contains antimicrobial, anti-inflammatory, and antioxidant compounds that enhance feed utilization, milk yield, and milk composition in ruminants [47]. The quantitative effects of MOLE on immune parameters across different ruminant species are summarized in Table 5.

In sheep, supplementation with aqueous MOLE in animals naturally co-infected with Fasciola gigantica and Clostridium novyi significantly altered serum immunoglobulin and cytokine levels, reducing pro-inflammatory markers while elevating the anti-inflammatory cytokine IL-10 [84].

In goats, dietary Moringa leaf powder (MOLP) fed during the periparturient period elevated plasma antioxidant capacity and reduced oxidative stress markers, alongside increases in immunoglobulins IgA, IgG, and IgM [85]. In early-weaned goat kids, supplementation with Moringa polysaccharides similarly increased serum immunoglobulins and antioxidant enzyme activities while decreasing MDA [86].

In dairy cows, feeding MOLM during the transition period increased colostrum IgG and reduced milk SCC compared to control animals, indicating improved udder health and reduced subclinical mastitis risk [47,48]. A separate study in lactating cows reported that MOLP supplementation lowered oxidative stress markers and improved milk quality, although statistical significance for immune parameters varied [87].

In calves, linear feeding of Moringa polysaccharides dose-dependently increased serum IgA, IgG, and IgM, with parallel improvements in CAT and T-AOC and a reduction in MDA [88].

Flavonoids are one of the most important bioactive components of MO that have the ability to promote immunity in animals due to their anti-pathogenic, anti-oxidation, and anti-inflammatory functions [83]. In bovine mammary epithelial (MAC-T) cells, MOLE reduced LPS-induced ROS production and suppressed the mRNA expression of pro-inflammatory cytokines IL-1β and TNF-α [85,89]. Other reports have shown that Moringa silages possess higher antioxidant capacity than fresh or dried leaves, likely due to the accumulation of low-molecular-weight amino acids and peptides during ensiling [13]. Furthermore, dietary MOL supplementation in ewes and lambs significantly enhanced hematological parameters and improved plasma profiles including total protein, albumin, globulin, glucose, urea, and minerals [90]. MOL also positively modulated gut microbiota by reducing opportunistic pathogens such as *Escherichia coli* and *Shigella* spp., while increasing beneficial *Lactobacillus* spp. [91,92].

Collectively, the moderate concentrations of phenolics and tannins in MOL contribute to its antioxidant and antimicrobial activities, with beneficial effects on the productive performance of ruminants [53]. Emerging evidence also indicates that nanoencapsulated MOLE and other novel formulations hold promise for managing subclinical mastitis and improving overall health in dairy animals [93].

The immunomodulatory actions of MOLE can be explained by the synergistic activity of its major bioactive components. Flavonoids (quercetin and kaempferol) and phenolic acids (chlorogenic acid, gallic acid) inhibit the NF-κB signaling pathway, thereby reducing the transcription of pro-inflammatory cytokines such as IL-1β, IL-2, IL-17, and TNF-α while promoting the expression of anti-inflammatory IL-10 [38,64]. Simultaneously, isothiocyanates present in MOLE activate the Nrf-2 antioxidant response element, upregulating endogenous antioxidant enzymes (catalase, superoxide dismutase, and glutathione peroxidase) and reducing oxidative stress markers like MDA [55,62]. The reduction in serum IgG observed in some studies does not indicate immunosuppression but rather reflects a decreased antigenic load due to antimicrobial effects against pathogens such as *Clostridium novyi* and *Escherichia coli* [84].

**Table 5:** Quantitative effects of MOLE/MOLP on immune and oxidative-stress parameters in ruminants.

Species	Supplement Form	Dose/Level	Parameter	% Change vs. Control	Citation
Sheep (co-infected)	Aqueous MOLE	Not specified	Serum IgG (light infection)	−28%	[84]
Sheep (co-infected)	Aqueous MOLE	Not specified	IL-2	−35%	[84]
Sheep (co-infected)	Aqueous MOLE	Not specified	IL-17	−30%	[84]
Sheep (co-infected)	Aqueous MOLE	Not specified	IL-10	+40%	[84]
Periparturient goats	MOLP	Not specified	Plasma T-AOC	+22%	[85]
Periparturient goats	MOLP	Not specified	CAT	+18%	[85]
Periparturient goats	MOLP	Not specified	MDA	−31%	[85]
Periparturient goats	MOLP	Not specified	IgA, IgG, IgM (range)	+15 to +25%	[85]
Early-weaned goat kids	Moringa polysaccharides	Not specified	Serum IgA	+26%	[86]
Early-weaned goat kids	Moringa polysaccharides	Not specified	Serum IgG	+31%	[86]
Early-weaned goat kids	Moringa polysaccharides	Not specified	Serum IgM	+19%	[86]
Early-weaned goat kids	Moringa polysaccharides	Not specified	CAT	+24%	[86]
Early-weaned goat kids	Moringa polysaccharides	Not specified	T-AOC	+29%	[86]
Early-weaned goat kids	Moringa polysaccharides	Not specified	MDA	−34%	[86]
Transition dairy cows	MOLM	16.66 g/100 kg BW	Colostrum IgG	+42%	[47]
Transition dairy cows	MOLM	16.66 g/100 kg BW	Milk SCC	−37%	[48]
Calves	Moringa polysaccharides	Linear dose	Serum IgA	+18%	[88]
Calves	Moringa polysaccharides	Linear dose	Serum IgG	+23%	[88]
Calves	Moringa polysaccharides	Linear dose	Serum IgM	+15%	[88]
MAC-T cells (in vitro)	MOLE	Not specified	LPS-induced ROS	−52%	[85,89]
MAC-T cells (in vitro)	MOLE	Not specified	IL-1β mRNA	−40 to −60%	[85,89]
MAC-T cells (in vitro)	MOLE	Not specified	TNF-α mRNA	−40 to −60%	[85,89]

## 6. Impact of MOLE on Rumen Microbiota and Fermentation

**Figure 2:**
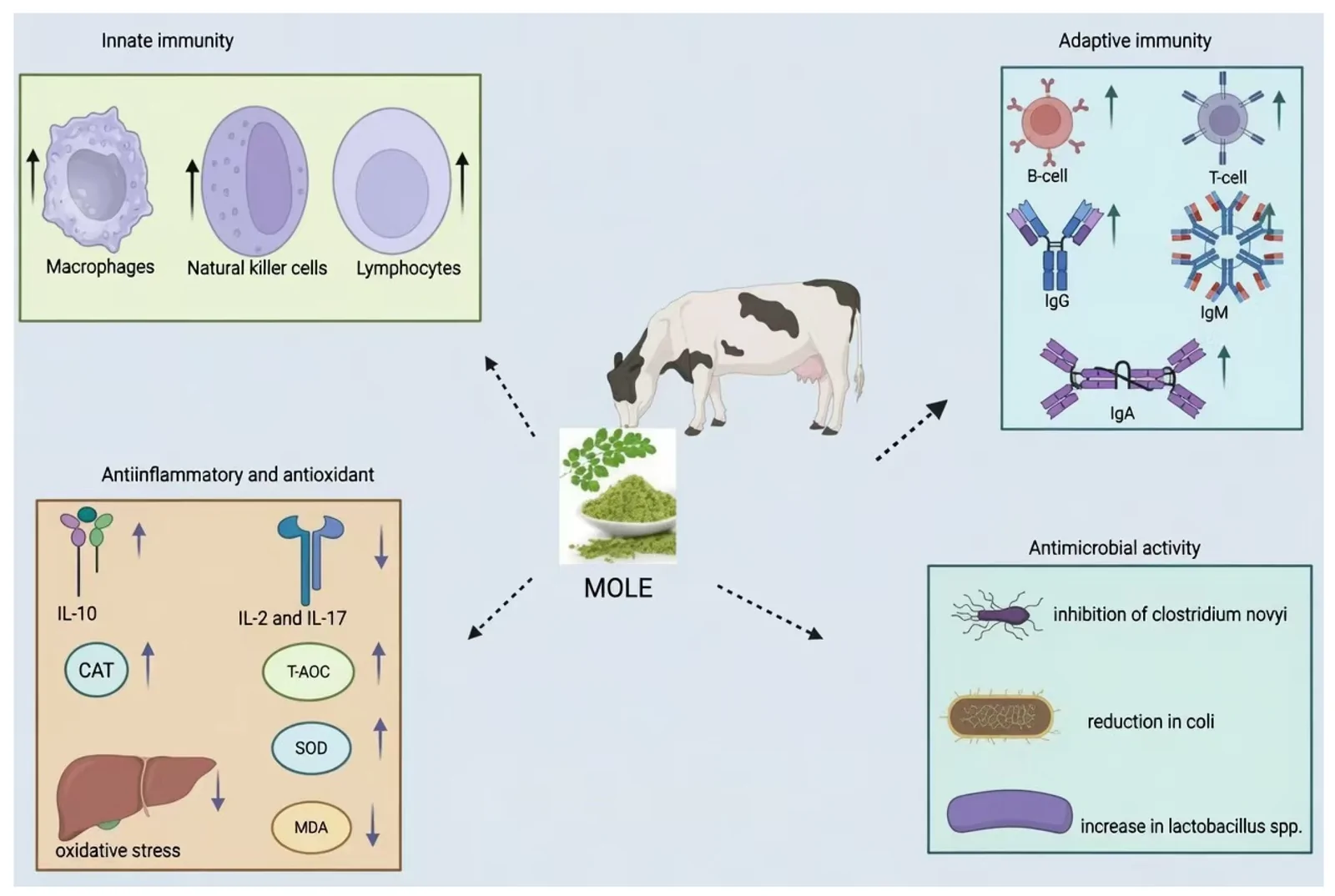
Schematic illustration of the pleiotropic effects of dietary MOLE on innate and adaptive immunity, antioxidant status, inflammation, and gut microbiota in livestock. The diagram depicts MOLE supplementation (central cow model representing ruminant or livestock intake) exerting modulatory influences (dashed arrows) across multiple physiological pathways. MOLE enhances innate immunity via activation of macrophages, natural killer cells, and lymphocytes. It stimulates adaptive immunity through B-cell and T-cell responses, leading to elevated production of immunoglobulins (IgG, IgM, and particularly IgA for mucosal protection. Arrows in the figure indicate the direction of change: solid arrows represent direct effects, dashed arrows represent modulatory influences, upward arrows indicate an increase, and downward arrows indicate a decrease.

Feeding MOL under normal conditions has been reported to improve growth performance, feed conversion ratio, and health status in several livestock species when fed at 5% or less of the total dry matter intake [94]. Dietary inclusion of MOLM significantly enhanced rumen fermentation by reducing key parameters such as ruminal enzyme activity, ruminal ammonia-nitrogen, total protozoan counts, and the acetate-to-propionate ratio, while increasing the concentrations of acetic acid, propionic acid, butyric acid, and total VFA [20] (Figure 2). Separately, supplementation with MO seeds combined with probiotics significantly lowered ruminal pH and dry matter degradability in both steers and sheep, and positively influenced methane production metrics [95]. Other findings indicated that while MO root bark proved less potent than monensin, it beneficially altered ruminal fermentation and improved nutrient digestibility [4]. In addition, one report demonstrated that feeding MOLP to kids significantly decreased ruminal levels of propionic acid, butyric acid, valeric acid, and ammonia nitrogen, while also significantly enhancing the height of rumen papillae [86]. Moreover, Moringa oil promoted the growth of *Prevotella* in both high- and low-roughage diets, which may help prevent or mediate rumen acidosis [54]. The major rumen fermentation and microbiota shifts reported in the literature are summarized in Table 6.

**Figure 3:**
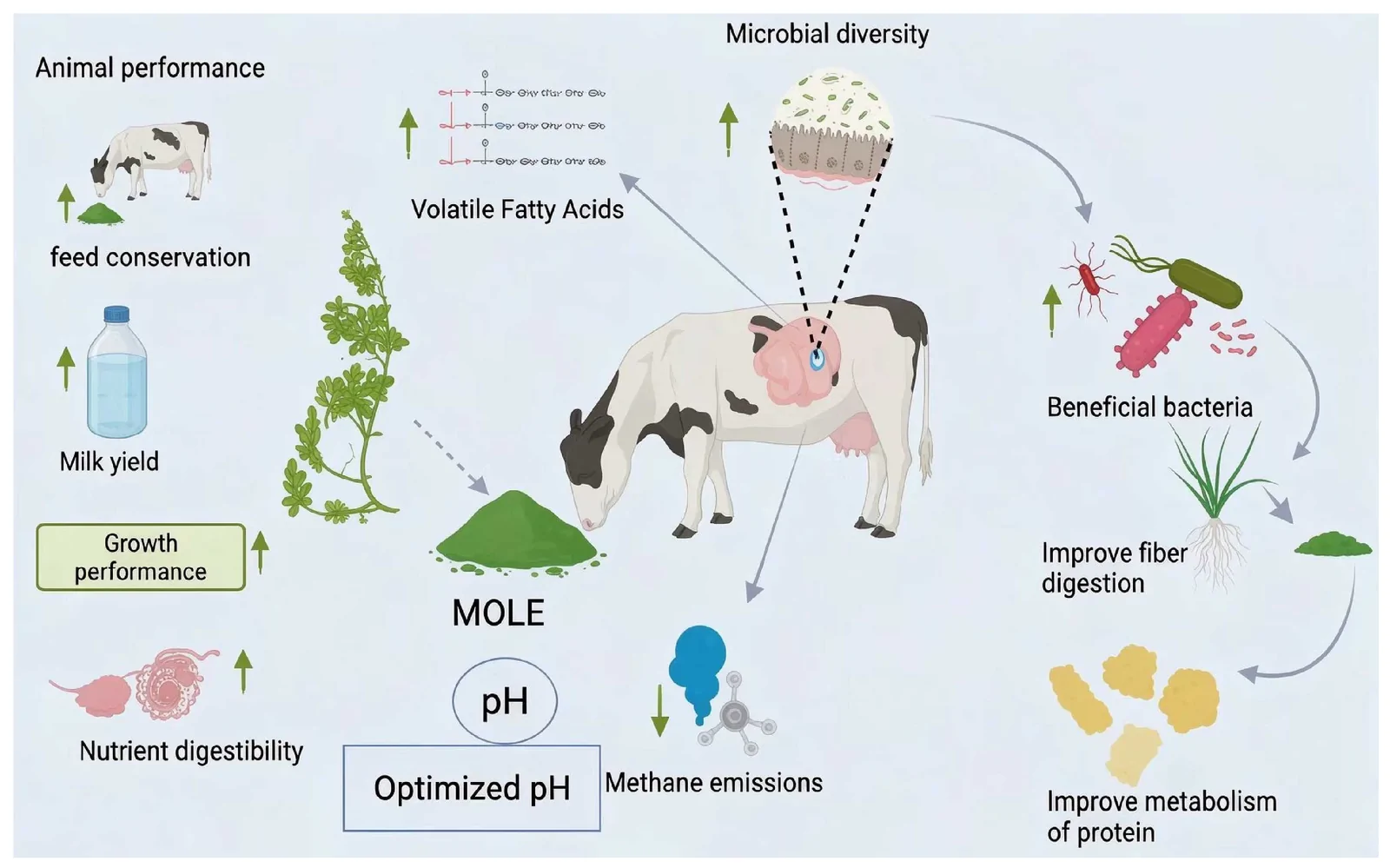
Schematic model illustrating the effects of MOLE supplementation on rumen fermentation dynamics, microbial ecology, VFA production, methane mitigation, nutrient utilization, and animal performance in ruminants. The central MOLE (green powder from Moringa leaves) acts as a phytogenic additive influencing ruminal processes in the depicted cow. MOLE optimize rumen pH (pH icon), promotes ↑ VFA; e.g., acetate, propionate, butyrate chemical structures), enhances microbial diversity and proliferation of beneficial bacteria (e.g., fiber-degraders and probiotic-like genera), and improves fiber digestion (plant root icons) and overall nutrient digestibility (protozoa/intestine icons). ↑ (Up Arrow): Indicates an increase, improvement, or promotion of the associated metric or biological process. ↓ (Down Arrow): Indicates a decrease, reduction, or inhibition of the associated metric or biological process. Solid Grey Arrows (→): Represent the causal relationship or pathway of influence, showing how one factor leads to or impacts another.

The MOLE group showed a decrease in microbial diversity with an increase in *Prevotella* and *Bacteroidales* populations, which have positive relationships with carbohydrate, protein, and VFA metabolism, and an increase in the proportions of *Treponema* sp., *Ruminococcus* sp., *Ruminobacter amylophilus*, and *Aeromonas*, which indicates better cellulose and nitrogen metabolism [96]. Similarly, animals fed with Moringa showed an increase in microbial genera related to VFA production (e.g., *Prevotella*, *Anaerovibrio*, *Lachnospiraceae*, *Butyrivibrio*, *Christensenella*) and those involved in starch and fiber digestion (e.g., *Proteobacteria*, *Ruminococcus*) [97]. Furthermore, MOL supplementation has proven effective in enhancing jejunal permeability and digestive function, positively altering microbiota composition, and boosting mucosal immunity in ruminants under heat stress [98]. Additionally, flavonoids can influence rumen metabolism and microbial fermentation (Figure 3). They also promote blood circulation in dairy cows, which improves overall metabolism, enhances nutrient absorption, and leads to increased milk yield [83]. Supplementation with MOLE as a feed additive in lactating ewes optimized ruminal pH and decreased estimated methane emissions, and also had a positive effect on rumen fermentation and microbial synthesis [99].

The observed modulation of rumen fermentation by MOLE can be explained by the selective antimicrobial activity of its phenolic and flavonoid compounds. These phytochemicals disrupt the cell membranes of sensitive microbes while sparing others, leading to a shift in community structure. The consistent reduction in protozoal counts [20] is particularly important because rumen protozoa are major contributors to methanogenesis through interspecies hydrogen transfer. By suppressing protozoa, MOLE reduces hydrogen availability for methanogens, thereby lowering methane production without directly inhibiting cellulolytic bacteria. The increase in cellulolytic genera such as *Ruminococcus* and *Treponema* and in VFA-producing bacteria like *Prevotella* and *Butyrivibrio* indicates that MOLE creates a more efficient fermentative environment. The reduction in ruminal ammonia-N suggests enhanced microbial protein synthesis or reduced proteolysis, possibly due to inhibition of hyper-ammonia-producing bacteria. The finding that Moringa oil promotes *Prevotella* [54] aligns with the high unsaturated fatty acid content of the oil, which may selectively enrich this genus.

**Table 6:** Rumen fermentation and microbiota shifts reported under MOLE/MOLM feeding.

Species	Supplement Form	Key Observed Changes	Citation
Buffalo calves	MOLM	↓ Ruminal enzyme activity, ↓ NH_3_-N, ↓ total protozoa, ↓ acetate:propionate; ↑ acetic, propionic, butyric acid, ↑ total VFA	[20]
Steers	MO seeds + probiotics	↓ Ruminal pH, ↓ DM degradability; ↓ CH_4_/ME, ↓ CH_4_/OM	[95]
Sheep	MO seeds + probiotics	↓ Ruminal pH, ↓ DM degradability; ↓ CH_4_/SCFA	[95]
Growing lambs	MO root bark	Beneficial alteration of ruminal fermentation, ↑ nutrient digestibility	[4]
Goat kids	MOLP	↓ Propionic acid, ↓ butyric acid, ↓ valeric acid, ↓ NH_3_-N; ↑ rumen papilla height	[86]
In vitro (high/low roughage)	Moringa oil	↑ Prevotella abundance	[54]
Lactating goats	MOL	↓ Microbial diversity; ↑ Prevotella, ↑ Bacteroidales, ↑ Treponema, ↑ Ruminococcus, ↑ R. amylophilus, ↑ Aeromonas	[96]
Small ruminants	MOL	↑ Prevotella, ↑ Anaerovibrio, ↑ Lachnospiraceae, ↑ Butyrivibrio, ↑ Christensenella, ↑ Proteobacteria, ↑ Ruminococcus	[97]
Lactating ewes	MOLE	Optimized ruminal pH, ↓ estimated CH_4_ emissions, positive effect on fermentation and microbial synthesis	[99]

↓ (Down Arrow): Indicates a decrease, reduction, or lower level of the specific parameter or metric following the MOLE/MOLM feeding intervention. ↑ (Up Arrow): Indicates an increase, elevation, or higher level of the specific parameter or metric following the MOLE/MOLM feeding intervention.

## 7. New Techniques for Extraction of MOLE Bioactive Components

**Figure 4:**
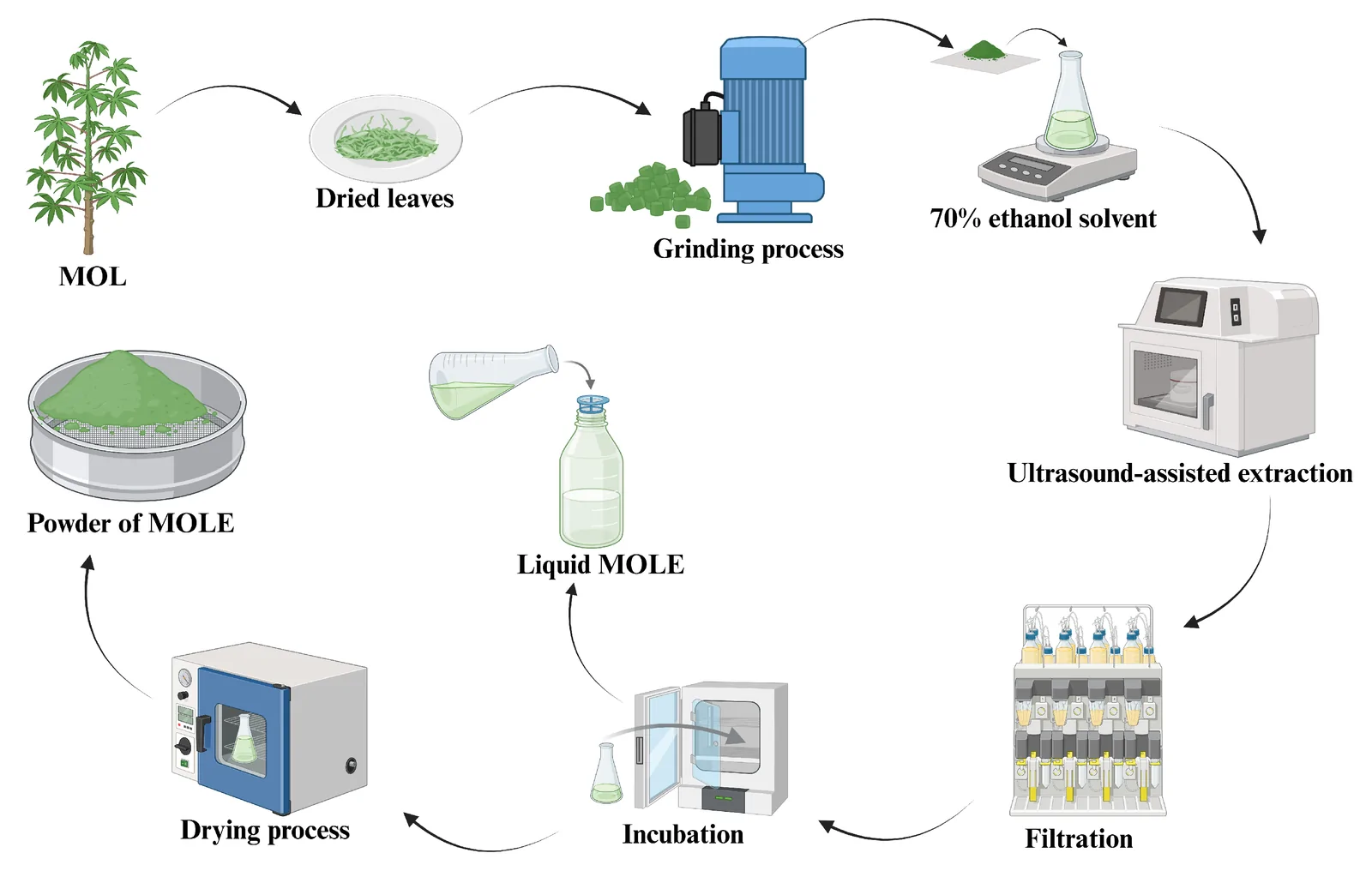
Schematic flowchart illustrating the preparation protocol for *MOL* Extract (MOLE) using Ultrasound-Assisted Extraction (UAE) with 70% ethanol as the solvent. The sequential steps are: (1) collection of fresh *MOL* leaves, (2) drying to obtain dried leaves, (3) grinding/milling to produce a fine powder, (4) addition of 70% ethanol solvent, (5) Ultrasound-Assisted Extraction (UAE) to release bioactive compounds (e.g., phenolics, flavonoids), (6) incubation to enhance mass transfer, (7) filtration to separate the liquid extract, and (8) drying (e.g., rotary evaporation or freeze-drying) to yield the final MOLE product, which can be obtained as either a liquid concentrate or a dried powder.

The moringa plant is exceptionally versatile and offers tremendous benefits, particularly in many impoverished regions of Africa, where its leaves serve as a vital dietary supplement to combat and prevent malnutrition [100]. It possesses outstanding nutritional, nutraceutical, and therapeutic value, largely due to its rich variety of bioactive compounds found across different parts of the plant, including proteins, flavonoids, saponins, phenolic acids, tannins, isothiocyanates, lipids, minerals, vitamins, and many others [45]. Due to being rich in active constituents, it also has many pharmacological activities including anti-oxidation, anti-inflammatory, anti-diabetic, lipid-lowering, anti-cancer and anti-bacterial activity [101]. The phenolic compounds and biological activities of MOL depend on the method and the solvent used for the extraction of these bioactive substances (Table 7) [102]. The interactions of these compounds with other food matrix components remain incompletely elucidated. Moreover, numerous extraction parameters including solvent composition, extraction duration, temperature, pH, solid-to-liquid ratio, and particle size can exert substantial influence on the efficiency and yield of solid–liquid extraction processes [103] (Figure 4). Traditional methods for extracting polyphenols from MOL, such as ethanol extraction, often face challenges like low efficiency, environmental concerns, and high operational costs [104]. Consequently, research has compared conventional solid–liquid extraction with techniques like ultrasound-assisted extraction (UAE), using various solvents and solvent–water mixtures [103]. Further studies have specifically investigated the phytochemical composition and extraction efficiency of MOL using four distinct methods: maceration, Soxhlet extraction, UAE, and microwave-assisted extraction (MAE), all utilizing a 70% hydroethanolic solvent [105,106].

While some previous studies used gas chromatography-mass spectrometry and found that between 28 and 34 different compounds can be extracted from MO using different extraction methods [107]. Other reports have shown that the Box–Behnken design (BBD) is one of the most frequently used methods for optimization [105]. Moreover, studies have found that UAE and MAE achieved higher oil recovery rates of 91-94% in significantly shorter extraction times compared to conventional extraction (CE), which yielded 90% [108]. Additionally, research has demonstrated that moringa oil can be successfully extracted on a pilot scale using supercritical fluid extraction (SFE) under relatively low temperature and pressure conditions, resulting in a high-quality oil product [109]. Another study highlighted Atmospheric Room Temperature Plasma (ARTP) as an innovative, non-thermal pretreatment technique. This method stands out as a promising green technology due to its low environmental impact, cost-effectiveness, and superior extraction efficiency relative to traditional approaches [110]. In further research, MOL were extracted using a 70% ethanol solution, followed by filtration and evaporation to dryness. The resulting dried MOLE was then employed to prepare a sodium alginate nanocomplex, utilizing calcium chloride (CaCl_2_) as the cross-linking agent through the ionic gelation method [111].

**Table 7:** Different new techniques used in extraction of bioactive components from different parts of MO.

Items	Extraction Methods	Part of Plant	Temperature	Solvent	References
Gallic acid	Maceration	MOL	40 °C	70% ethanol	[112]
Flavonoids, alkaloids	microwave-assisted extraction	MO flowers		Eutectic Solvents	[113]
Yield	soxhlet extraction	MOL powder	Room temperature	70% ethanol	[70]
Polysaccharides	enzyme-assisted extraction	MOL			[114]
Leaf extracts	Ultrasound-assisted extraction method	MOL powder	Less than 30 °C	70% ethanol	[105]
Phenolic compounds	Hot bath and vacuum pressure	MOL	60 °C	Ethyl acetate	[115]
Phenolic compounds	Ultra-high-pressure liquid Chromatography	MOL		Methanol 100%	[100]
Flavonoid	successive Maceration	MOL		Dichloromethane	[102]
Tannic acid	Maceration and ultrasonication	MOL powder	Room temperature	Phenol reagent and sodium carbonate	[116]
Flavonoids and phenolic compounds	Maceration and decoction	dried leaves	100 °C	Boiling distilled water	[117]

## 8. Advances in Nanoencapsulation and Formulation Technologies of MOLE

Microencapsulation is an emerging technology extensively applied in animal nutrition to create stable forms of vitamins, minerals, and fatty acids [21]. For example, MOLE has been successfully encapsulated into composite ultrafine nanoparticles consisting of poly-ε-caprolactone (PCL) and montmorillonite (MMT), referred to as PCL-loaded MO/MMT NPs, through a salting-out technique (Table 8). Currently, polysaccharides and proteins are the most commonly used materials for encapsulation, followed by lipids. Among recent encapsulation techniques are spray drying, cross-linking gelation, freeze-drying, nanoencapsulation, electrospinning, and electrospraying [118]. Ionic gelation stands out as a particularly attractive chemical microencapsulation approach because it is inexpensive, straightforward, and practical, requiring neither organic solvents nor high-temperature processing. In this method, alginate is widely favored as a coating material owing to its low cost, excellent biocompatibility, non-toxicity, and strong gelling and stabilizing properties [119].

Polysaccharides and proteins remain the primary encapsulation materials (followed by lipids), with emerging methods including spray drying, cross-linking gelation, freeze-drying, nanoencapsulation, electrospinning, and electrospraying. Importantly, such encapsulation strategies can help mitigate potential cytotoxicity concerns associated with certain MO compounds [118].

Regarding the biological effects of nanoencapsulated MOLE in dairy ruminants, emerging evidence indicates that encapsulation can enhance or prolong the bioactive properties of the extract. For instance, nanoencapsulated MOLE administered to rabbit does during summer (a model for heat stress) alleviated disruptions in metabolism, redox status, and hormonal balance. These beneficial effects were observed with both free and encapsulated forms, but nanoencapsulation improved stability and sustained release [120]. In the context of rumen fermentation and methane mitigation, nanoencapsulation of MOLE has been shown to reduce ruminal methane, carbon monoxide, and hydrogen sulfide outputs when added to high-concentrate diets in vitro, suggesting that encapsulation preserves the anti-methanogenic activity of MOLE while potentially protecting bioactive compounds from rumen degradation [21]. Although direct studies on productive parameters, immunity, and milk composition in dairy ruminants using nanoencapsulated MOLE are still limited, the available data support that nanoencapsulation enhances the bioavailability and targeted delivery of Moringa bioactive compounds. This technology may therefore amplify the positive effects of MOLE on feed efficiency and immune modulation (such as reduced oxidative stress and inflammatory markers), milk yield and composition (such as increased fat and protein and reduced SCC), and rumen microbiota modulation (such as increased cellulolytic bacteria and reduced protozoa). Future research should specifically evaluate nanoencapsulated MOLE in lactating dairy cows and goats to quantify these potential improvements and establish optimal encapsulation protocols for commercial application [93,95].

Furthermore, the addition of MOLE has been shown to alleviate the adverse effects of heat stress, such as disruptions in metabolism, redox status, and hormonal balance, during pregnancy. These beneficial effects were observed irrespective of whether the MOLE was administered in its free form or in an encapsulated form [120].

**Table 8:** The nanoencapsulation of MO extraction and their application in daily life.

Encapsulation Technique	Encapsulated Material	Encapsulation Matrix	Application	References
Cross-linking gelation	MO seed powder	Sodium alginate	Pollutants removal	[118]
Microencapsulation	Phenolics		Anti-diabetic	[121]
Freeze dryer	MO seeds		Antioxidant	[122]
Mono nuclear phagocyte system (MPS) cells	MOLE		Antimicrobial	[123]
Nanoencapsulation	Oleic acid methyl ester	Sodium tripolyphosphate	Reduce ruminal methane	[21]
Cross-linking gelation	Polysaccharides and proteins		reduced oxidative degradation	[124]
Novel formulation techniques	Flavonoids and phenolic		Improve patient compliance	[125]
‘Green’ nanoparticles	MOLE		Anti-diabetic	[126]
Metal oxide nanoparticles	MOLE		Nutritional	[127]
Ionic gelation	Phenolic compound	Sodium alginate	Antioxidant	[119]

## 9. Research Bottlenecks and Future Directions

The roadmap for the continued development of MOLE research and applications points to important areas in agriculture, clinical standardization, green nanotechnology, and sustainability policy. However, while each of them holds immense promise, well-thought-out strategies are needed to overcome the challenges at hand and promote inclusive and evidence-based progress [128]. The medicinal use of MOLE is still limited and poorly advertised, especially regarding registered clinical trials for MOL-related studies [129]. Additionally, the safety and toxicity profile of products derived from the moringa plant has been thoroughly investigated through extensive in vitro and in vivo studies, as well as a substantial number of human clinical trials. These efforts have aimed to validate its therapeutic potential across various medical conditions and clinical scenarios.

Nevertheless, despite this robust body of evidence combined with the plant’s inherent advantages, such as its widespread global availability and minimal cultivation requirements, the translation of MOLE’s promising biological properties into routine clinical practice remains limited. This is clearly reflected in the scarcity of approved or registered clinical trials specifically focused on this plant [130].

In summary, researchers continue to explore and harness the multifaceted value of the moringa plant across diverse fields, including agriculture, biology, and food science. Particular emphasis is placed on its pharmacological, nutritional, and biofuel applications, with the overarching goal of maximizing its contributions to human health, environmental sustainability, and long-term development [130,131]. Additionally, future directions should focus on the development of green MOL and sustainable extraction technologies. While new extraction methods have recently been used, including microwave-assisted extraction and ultrasound-assisted extraction, advanced techniques such as supercritical fluid extraction with CO_2_ (modified to decrease their ecological impact) and pressurized liquid extraction should also be considered. All extraction methods should have higher selectivity, better preservation of thermolabile compounds, and reduced environmental impact. Additionally, more comparative studies to evaluate the efficacy, environmental impact and cost of these advanced methods are needed to establish industry standards. Furthermore, research into sequential or integrated extraction processes that fractionate different bioactive classes, such as proteins, phenolics, and sugars, from the same biomass in a zero-waste paradigm is a crucial gap. Finally, no meta-analysis has been published to date that quantitatively synthesizes the effects of MOLE across comparable outcomes, largely due to high heterogeneity in extraction protocols, dosage forms, and study designs. Moreover, long-term controlled trials in animal models or humans are conspicuously absent, and most studies report outcomes over just weeks to months, leaving questions about sustained efficacy, safety, and toxicity.

## 10. Limitations of the Review

While this is a comprehensive review of the literature for the biological activities and nutritional uses of MOLE, some limitations should be noted. First, three major databases (PubMed, Scopus and Google Scholar) were searched, so some additional pertinent studies may not have been identified in other databases. Secondly, all articles were in English only, causing potential language bias and excluding potentially useful articles in other languages.

Critically, no multi-dose titration studies have been conducted to establish dose–response relationships for MOLE across ruminant species and production stages. Such studies—using standardized extraction protocols and uniform dosing units—are urgently needed to generate the practical recommendations that producers and nutritionists require. We intend to address this gap in a future systematic review and meta-analysis, once sufficient dose–response data become available in the peer-reviewed literature.

Additionally, this review was not conducted in a systematic manner, including independent screening by multiple reviewers, calculation of interrater agreement, or quantitative risk of bias assessment of studies included. Thus, the results should be regarded as a complete narrative synthesis, and not as a systematic review or meta-analysis. Furthermore, the variety of animal species, experimental conditions, extraction procedures, doses, and supplementation times in the various studies may contribute to variations in results and make it difficult to directly compare studies.

### Testable Hypotheses for Future Mechanistic Work

The following hypotheses emerge from the patterns observed in the reviewed literature but have not yet been directly tested. They are presented here to guide future mechanistic research.

(a) Bioactive isothiocyanates in MOLE specifically inhibit Butyrivibrio and other biohydrogenating bacteria, leading to an increased flow of unsaturated fatty acids to the mammary gland. This hypothesis could be tested via metatranscriptomic analysis of rumen microbiota from MOLE-supplemented animals.
(b) Phenolic compounds in MOLE act as electron sinks, diverting electrons away from methanogenesis toward alternative pathways such as propionate synthesis. This is supported by the observed decrease in the acetate:propionate ratio [20] and warrants confirmation through in vitro hydrogen-balance experiments.
(c) Isothiocyanates in MOLE specifically suppress Methanobrevibacter species while having minimal effect on fibrolytic bacteria. Future research should employ metagenomic sequencing to identify the precise microbial genes and pathways modulated by MOLE.
(d) The immunomodulatory outcome of MOLE is dose-dependent: lower doses may preferentially enhance innate immunity (increased phagocytosis, NK cell activity), while higher doses suppress excessive inflammation (reduced Th17 response). This dual action would explain the simultaneous rise in IgA (mucosal immunity) and reduction in systemic IL-17 observed in supplemented ruminants. Future studies should explore the threshold concentrations at which MOLE switches from immunostimulatory to anti-inflammatory, as this would allow targeted use for either disease prevention or management of chronic inflammatory conditions.

## 11. Conclusions

To summarize, MOLE rich in flavonoids and phenolic compounds has been shown to be able to effectively modulate rumen microbial ecology, increasing digestibility and production of the volatile fatty acids, and having anti-inflammatory and antioxidant effects that bolster immunity and decrease disease incidence. In addition, it has a positive effect on milk production and quality, and enhances beneficial fat and protein profiles in milk. Furthermore, a comprehensive dairy industry adoption of these benefits will require future research to be strongly driven by the importance of developing a standardized extraction protocol for the production of bioactive compounds and to define precisely optimal dosage rates by production stage. Critically, comprehensive long-term safety assessments, including residue analyses and chronic toxicity evaluations alongside robust multi-herd validation under real-world commercial farming conditions, are imperative to confirm sustained efficacy, ensure animal well-being across complete production cycles, and satisfy regulatory requirements for feed additive approval, ultimately paving the way for MOLE responsible and scalable implementation in sustainable ruminant production systems.

## Data Availability

The original contributions presented in this study are included in the article. Further inquiries can be directed to the corresponding authors.

## Abbreviations

MO	*Moringa oleifera*
MOLE	*Moringa oleifera* Leaf Extract
MOL	*Moringa oleifera* Leaves
MOLM	*Moringa oleifera* Leaf Meal
MOLP	*Moringa oleifera* Leaf Powder
MOML	*Moringa oleifera* Leaf Extract (methanolic/ethanol)
DM	Dry Matter
BW	Body Weight
VFA	Volatile Fatty Acids
SCC	Somatic Cell Count
IgG, IgA, IgM	Immunoglobulin G, A, M
IL-1β, IL-2, IL-10, IL-17	Interleukin-1 beta, 2, 10, 17
TNF-α	Tumor Necrosis Factor-alpha
ROS	Reactive Oxygen Species
MDA	Malondialdehyde
T-AOC	Total Antioxidant Capacity
CAT	Catalase
SOD	Superoxide Dismutase
GSH-Px	Glutathione Peroxidase
NF-κB	Nuclear Factor Kappa B
Nrf-2	Nuclear factor erythroid 2-related factor 2
COX	Cyclooxygenase
LOX	Lipoxygenase
NO	Nitric Oxide
PGE2	Prostaglandin E2
ERK	Extracellular signal-Regulated Kinase
MEK	Mitogen-Activated Protein Kinase Kinase
CYP450	Cytochrome P450
GABA	Gamma-Aminobutyric Acid
MAC-T	Bovine Mammary Epithelial Cell line
LPS	Lipopolysaccharide
UAE	Ultrasound-Assisted Extraction
MAE	Microwave-Assisted Extraction
CE	Conventional Extraction
SFE	Super-controlled Fluid Extraction (supercritical CO_2_)
ARTP	Atmospheric Room Temperature Plasma
BBD	Box–Behnken Design
PCL	Poly-ε-caprolactone
MMT	Montmorillonite
NPs	Nanoparticles
PCL-MO/MMT NPs	PCL-loaded MOLE/MMT nanoparticles
CaCl_2_	Calcium Chloride
MPS	Mononuclear Phagocyte System
DPPH	Diphenyl-1-picrylhydrazyl
HPLC	High-Performance Liquid Chromatography
GC-MS	Gas Chromatography–Mass Spectrometry
OM	Organic Matter
SCFA	Short-Chain Fatty Acids
CH_4_	Methane
CO_2_	Carbon Dioxide
H_2_S	Hydrogen Sulfide
RBC/WBC	Red/White Blood Cells
ALT/AST	Alanine/Aspartate Transaminase (Glutamic/Pyruvic transaminase)
N-NH_3_/NH_3_-N	Ammonia Nitrogen
ECM	Energy-Corrected Milk
DIM	Days In Milk
SEM	Standard Error of Mean
